# Tuberculosis in renal transplant recipient

**DOI:** 10.4103/0971-4065.73436

**Published:** 2010-10

**Authors:** P. Kalita, C. Phukan, S. Ali, A. K. Barman

**Affiliations:** Department of Medicine, Gauhati Medical College and Hospital, Guwahati-32, Assam, India; 1 Department of Nephrology, Gauhati Medical College and Hospital, Guwahati-32, Assam, India

Sir,

We read with interest the article on the allograft and prostatic involvement in a renal transplant recipient with disseminated tuberculosis by Sreejith *et al*.[1] We have also encountered a few patients of renal transplant with tuberculosis. However, we have a few queries.

- Is culture and sensitivity necessary to all patients with tuberculosis? It has been found that primary drug resistance is increasingly being reported in the general population.[2]
- What is the ideal duration of treatment for such patients? The regimens used in different centers vary in dose and duration. These regimens are not validated and are based on individual preferences and responses.[2]
- Will transrectal ultrasonography (TRUS) guided aspiration of prostatic abscess be an ideal treatment in a situation like this? TRUS guided aspiration of prostatic abscess is a safe and effective method.[3] It has proven its efficacy even in tubercular prostatic abscess.[4]
